# Automated image analysis of NSCLC biopsies to predict response to anti-PD-L1 therapy

**DOI:** 10.1186/s40425-019-0589-x

**Published:** 2019-05-06

**Authors:** Sonja Althammer, Tze Heng Tan, Andreas Spitzmüller, Lorenz Rognoni, Tobias Wiestler, Thomas Herz, Moritz Widmaier, Marlon C. Rebelatto, Helene Kaplon, Diane Damotte, Marco Alifano, Scott A. Hammond, Marie-Caroline Dieu-Nosjean, Koustubh Ranade, Guenter Schmidt, Brandon W. Higgs, Keith E. Steele

**Affiliations:** 1 ONE LOGIC, Munich, Germany; 2 Definiens, Munich, Germany; 3 AstraZeneca, Gaithersburg, MD 20878 USA; 4 INSERM, UMR 1138, Cordeliers Research Center (CRC), Paris, France; 5 Paris Descartes University (Sorbonne Paris Cité University), UMRS 1138, CRC, Paris, France; 6 Assistance Publique-Hôpitaux de Paris (AP-HP), Department of Pathology, Cochin Hospital, Paris, France; 7 Assistance Publique-Hôpitaux de Paris (AP-HP), Department of Thoracic Surgery, Cochin Hospital, Paris, France; 8 Sorbonne University, UMRS CR7, INSERM U1135 - CNRS ERL 8255, Centre d’Immunologie et des Maladies Infectieuses, Paris, France; 9 Immunocore LLC, Conshohocken, PA 19428 USA

**Keywords:** Biomarker, Cancer immune checkpoint therapy, CD8, Image analysis, Immunohistochemistry, NSCLC, PD-L1

## Abstract

**Background:**

Immune checkpoint therapies (ICTs) targeting the programmed cell death-1 (PD1)/programmed cell death ligand-1 (PD-L1) pathway have improved outcomes for patients with non-small cell lung cancer (NSCLC), particularly those with high PD-L1 expression. However, the predictive value of manual PD-L1 scoring is imperfect and alternative measures are needed. We report an automated image analysis solution to determine the predictive and prognostic values of the product of PD-L1+ cell and CD8+ tumor infiltrating lymphocyte (TIL) densities (CD8xPD-L1 signature) in baseline tumor biopsies.

**Methods:**

Archival or fresh tumor biopsies were analyzed for PD-L1 and CD8 expression by immunohistochemistry. Samples were collected from 163 patients in Study 1108/NCT01693562, a Phase 1/2 trial to evaluate durvalumab across multiple tumor types, including NSCLC, and a separate cohort of 199 non-ICT- patients. Digital images were automatically scored for PD-L1+ and CD8+ cell densities using customized algorithms applied with Developer XD™ 2.7 software.

**Results:**

For patients who received durvalumab, median overall survival (OS) was 21.0 months for CD8xPD-L1 signature-positive patients and 7.8 months for signature-negative patients (*p* = 0.00002). The CD8xPD-L1 signature provided greater stratification of OS than high densities of CD8+ cells, high densities of PD-L1+ cells, or manually assessed tumor cell PD-L1 expression ≥25%. The CD8xPD-L1 signature did not stratify OS in non-ICT patients, although a high density of CD8+ cells was associated with higher median OS (high: 67 months; low: 39.5 months, *p* = 0.0009) in this group.

**Conclusions:**

An automated CD8xPD-L1 signature may help to identify NSCLC patients with improved response to durvalumab therapy. Our data also support the prognostic value of CD8+ TILS in NSCLC patients who do not receive ICT.

**Trial registration:**

ClinicalTrials.gov identifier: NCT01693562.

Study code: CD-ON-MEDI4736-1108.

Interventional study (ongoing but not currently recruiting).

Actual study start date: August 29, 2012.

Primary completion date: June 23, 2017 (final data collection date for primary outcome measure).

**Electronic supplementary material:**

The online version of this article (10.1186/s40425-019-0589-x) contains supplementary material, which is available to authorized users.

## Background

The immune response to cancer is complex and involves a number of cellular proteins that may promote or suppress immune function. Interaction between programmed cell death ligand-1 (PD-L1) and its receptor programmed cell death-1 (PD1), which is expressed primarily on T lymphocytes, exemplifies a major immunosuppressive pathway [1, 2]. PD1 signaling interferes with T-lymphocyte activation and can result in T-cell anergy or lymphocyte apoptosis. Multiple cancer types, including non-small cell lung cancer (NSCLC), exploit this pathway through expression of PD-L1 on neoplastic cells or immune cells, primarily macrophages. PD-L1 interacts with PD1+ cells, downregulating the tumoricidal activity of tumor infiltrating lymphocytes (TILs). Immune checkpoint therapy (ICT) targeting the PD1/PD-L1 pathway has greatly improved survival for NSCLC patients [3–7], leading to drug approvals across several countries. Despite recent successes, many patients treated with these antibodies fail to respond. As a result, multiple approaches to predict patient response to anti-PD1/PD-L1 therapies have been studied in recent years in the expanding field of precision medicine.

PD-L1 expression assessed by immunohistochemistry (IHC) has been at the forefront of predictive biomarkers for ICT. Higher PD-L1 expression on tumor cells (TCs) and/or immune cells has been associated with greater efficacy of anti-PD1/PD-L1 immunotherapies [4, 6–13]. Multiple PD-L1 IHC assays with various cutoff values have been developed commercially and are approved for companion or complementary diagnostic use [14]. As such, PD-L1 IHC assays currently represent the benchmark for predicting response to PD1/PD-L1 blockade. However, their clinical utility has been questioned, as some PD-L1-low/negative patients show a therapeutic response and some PD-L1-high patients fail to respond [15]. Durvalumab is a selective, high-affinity, engineered human IgG1 monoclonal antibody that blocks PD-L1 binding to PD1 and CD80 [16]. Consistent with other immunotherapies targeting the PD1/PD-L1 axis, greater response rates and longer survival have been observed in durvalumab-treated NSCLC patients with biopsy specimens that express ≥25% membranous PD-L1 TC compared to those with < 25% PD-L1 TC. This was shown in two separate trials: a nonrandomized Phase 1/2 trial evaluating durvalumab in advanced NSCLC and other solid tumors (Study 1108/NCT01693562) and PACIFIC, a randomized Phase 3 trial of durvalumab after chemoradiotherapy in stage III, locally advanced, unresectable NSCLC (NCT02125461) [7, 10]. Notably, in both trials, responses were observed in a proportion of patients with < 25% PD-L1.

Given the limitations of PD-L1 IHC, alternative diagnostic strategies have been studied to find better predictors of response to PD1/PD-L1 blockade. Key among these is tumor mutational burden (TMB), which has recently been shown to have promising predictive potential for anti-PD1/PD-L1 monotherapy and combination therapy with anti-cytotoxic T-lymphocyte-associated protein 4 agents in multiple NSCLC clinical settings [17–22]. Likewise, we and others have shown that interferon-γ gene-related signatures are associated with improved response to durvalumab, atezolizumab, and nivolumab [6, 23, 24]. The number of CD8+ TILs in the tumor microenvironment also has predictive potential in NSCLC [25, 26]. However, these tumor-based methods have limitations similar to those of PD-L1 IHC assays as discussed above. Separately, the presence or absence of liver metastases, as well as measures of circulating tumor cell DNA, have also been investigated as predictors of response to anti-PD1/PD-L1 therapy in NSCLC [27, 28].

Relevant to assessment of the predictive potential of a cancer therapy screening test is the need to assess its prognostic value, especially with respect to standard of care chemotherapy. A recent literature review, and other studies, have found conflicting results of the prognostic value of PD-L1 expression in NSCLC patients generally or those receiving chemotherapy [6, 29–31]. These studies included measurements of PD-L1 by IHC as well as by cytometry and gene expression. In contrast to PD-L1, multiple CD8 measures have more consistently demonstrated that CD8+ TIL densities are associated with longer survival in NSCLC patients who are not receiving ICT [32–35].

To better identify patients likely to derive clinical benefit from anti-PD-L1 immunotherapy, we explored a biomarker signature consisting of tumoral CD8+ cell density (TILs/mm^2^ of tumor) multiplied by PD-L1+ cell density using automated image analysis (CD8xPD-L1 signature). We chose a digital approach to quantify CD8+ and PD-L1+ cell densities, as properly validated image analysis solutions have multiple advantages over manual assessment. These include the production of continuous quantitative data, improved reproducibility and avoidance of inter-observer variability, which is an inherent disadvantage of manual assessments of both PD-L1 and CD8 expression [36, 37]. The main goal of our study was to determine whether the CD8xPD-L1 signature better predicts response to durvalumab compared with the signature’s two individual components and with manual PD-L1 scoring. The CD8xPD-L1 signature, its two components, and manual PD-L1 scoring were also investigated for possible associations with patient survival in the non-ICT setting using an independent data set of NSCLC patients receiving standard of care treatment.

## Methods

### Patient cohorts

To assess the predictive potential of the CD8xPD-L1 signature, baseline archival or fresh tumor samples were analyzed from NSCLC patients enrolled in Study 1108/NCT01693562. The primary endpoints were the safety profile across various tumor types and antitumor activity of durvalumab in NSCLC and urothelial carcinoma. Secondary study endpoints were assessment of antitumor activity for all other investigated tumor types, as well as pharmacokinetics and immunogenicity. Assessments of antitumor activity included objective response rate (ORR), disease control rate, duration of response, and progression-free survival (PFS) using Response Evaluation Criteria in Solid Tumors (RECIST) version 1.1 guidelines [38], as well as overall survival (OS). Eligible patients had histologically or cytologically confirmed advanced squamous or non-squamous NSCLC and had failed, were intolerant of, ineligible for, or had refused an approved first-line treatment. They were required to be ≥18 years old and have an Eastern Cooperative Oncology Group performance status of 0 or 1, with adequate organ and marrow function. All participants provided written informed consent before undergoing study procedures. This study was conducted in accordance with the Declaration of Helsinki and Good Clinical Practice guidelines. The clinical protocol for this study was approved by appropriate institutional review boards and ethics committees.

Patients in Study 1108 were initially enrolled regardless of tumor PD-L1 expression. Tumor samples from these patients were used to develop an IHC assay to determine PD-L1 expression (SP263 assay [Ventana Medical Systems, Inc.]) [39]. After assay validation, subsequent patients were screened for PD-L1 expression and protocol amendments enriching for PD-L1 expression ≥25% began in June 2013. This cutoff was chosen based on the population prevalence of PD-L1 expression, ease of scoring, maximizing negative predictive value and differentiating responders from non-responders [39].

Baseline tumor specimens with consecutive slides of CD8 and PD-L1 stains were available for 163 patients from Study 1108. These were split between a training set (*n* = 84) and a test set (*n* = 79), which were balanced by PD-L1 status (PD-L1 ≥ 25%), ORR, previous lines of therapy, stage, and gender. To understand whether the CD8xPD-L1 signature differed in patients with NSCLC who had not been treated with a checkpoint inhibitor, an additional 199 surgically resected baseline specimens were analyzed from an independent cohort of non-ICT-treated patients who underwent surgery between 2001 and 2005. Patients with advanced-stage NSCLC received cisplatin + gemcitabine (approximately 50% of cases), cisplatin + vinorelbine, cisplatin + paclitaxel, or platinum salt/other drugs. For stage I disease, all patients underwent surgery and rarely received adjuvant chemotherapy. For stage II–IIIA disease, patients underwent surgery followed by adjuvant chemotherapy. For stage IIIB disease, patients received neo-adjuvant chemotherapy or if they did not respond to chemotherapy, they underwent radiotherapy, followed by surgery, followed by adjuvant chemotherapy. Baseline patient characteristics from both sample cohorts are shown in Additional file 2: Table S1.

### Immunohistochemistry

4-μm histological sections were prepared from formalin-fixed, paraffin-embedded tumors and mounted on positively-charged glass slides. Baseline tumor biopsies from Study 1108/NCT01693562 were immunostained separately for PD-L1 (clone SP263, Ventana Medical Systems, Inc., Tucson, AZ, USA) and for CD8 (clone SP239, Spring Bioscience, Pleasanton, CA, USA), both performed on the Ventana BenchMark ULTRA staining platform (Ventana Medical Systems, Inc., Tucson, AZ, USA) [37, 39]. For the non-ICT patient specimens, a CD8/PD-L1 dual immunostain using these antibodies was applied. All immunostained slides were digitally scanned and the image files were uploaded for digital processing as previously described [37].

### Image analysis

**Fig. 1:**
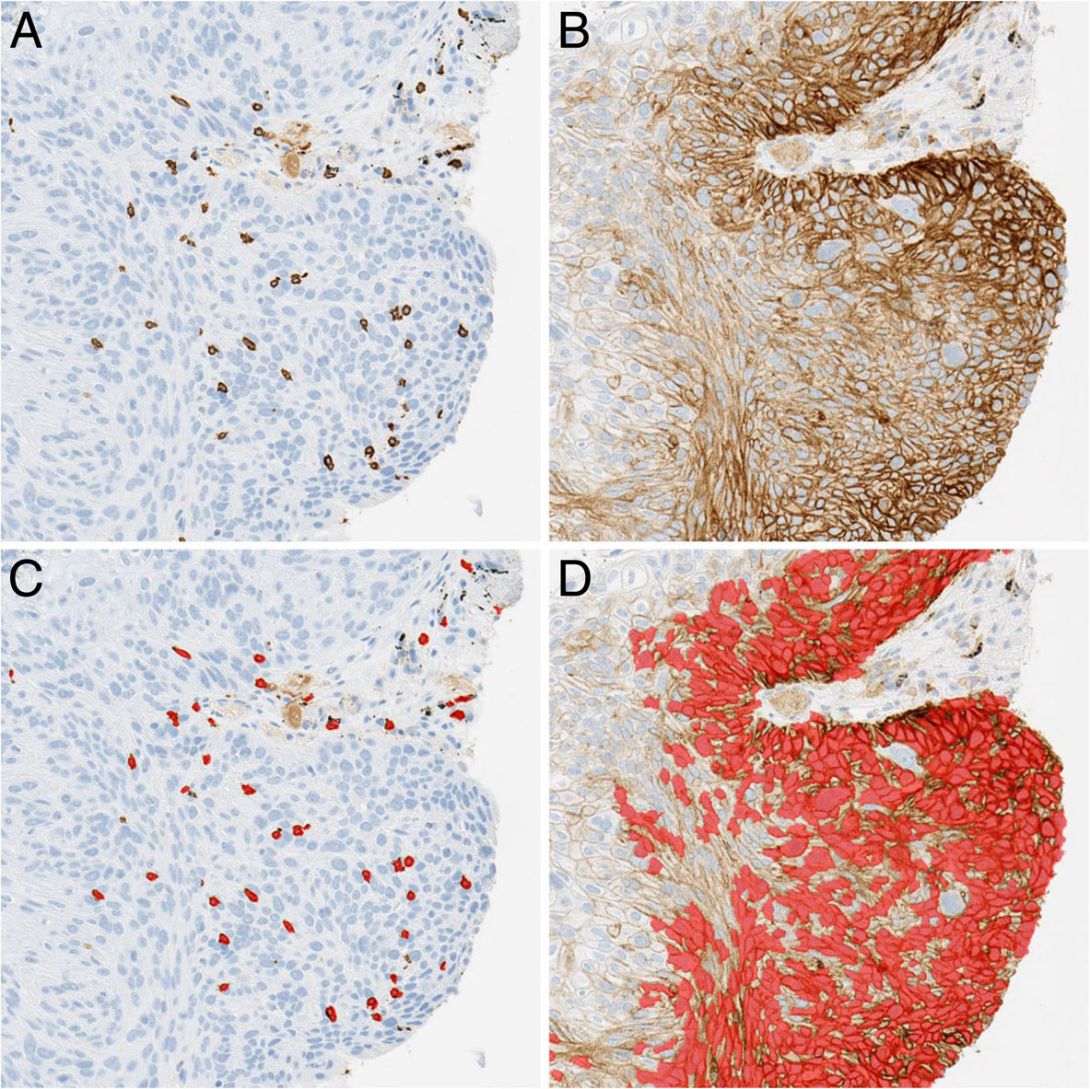
Digital image analysis segmentation of CD8+ and programmed cell death ligand-1 (PD-L1) + cells in single immunohistochemistry labelled sections of non-small cell lung cancer. Serial tumor sections of durvalumab-treated patients enrolled in Study 1108 were labelled separately using brown chromogen for both CD8 (**a**) and PD-L1 (**b**). Image analysis segmentations of cells expressing each marker (**c** and **d**) are shown as red and quantifications of the corresponding expression levels are performed separately

Rule-based methods combined with machine learning were used to segment and classify cells and nuclei of acquired images [40] using Developer XD™ 2.7 software (Definiens AG, Munich, Germany). The pathologists’ expert knowledge was translated into automated image analysis solutions to detect CD8+ and PD-L1+ cells in single marker images as shown in Fig. 1, or multiplex IHC images (Additional file 1: Figure S1). Image variability caused by histological quality and immunostaining variability across different samples was accounted for by unmixing the three-color red-green-blue image into marker-specific colors (brown-blue/purple-brown-blue) before analysis (Additional file 1: Figure S2). The readouts used for this study were based on positive cells that were detected in pathologist-annotated tumor regions as previously described [37]. To compare readouts across samples, the data were normalized by area (cells/mm^2^). All digital images were manually reviewed to ensure the quality of immunostaining, digital scanning and the precise detection of positive cells by image analysis. In addition, the quality of signal detection for both PD-L1 and CD8 in single- and dual-stain assays was validated as comparable, as previously reported [37]. For PD-L1, the percentage of TCs demonstrating membranous immunolabeling at any intensity was also assessed microscopically by a trained pathologist using a pre-determined cutoff of ≥25% for high PD-L1 expression [39]. The cutoff values for CD8 and PD-L1 measures by image analysis were determined separately by optimization in terms of positive predictive value (PPV) on the training set (see Statistical analysis), resulting in categorical variables that were used for the multivariate Cox regression analysis. For high density, cutoff values were established as 297 cells/mm^2^ for CD8+ cells and 644 cells/mm^2^ for PD-L1+ cells (tumor cells + macrophages). The CD8xPD-L1 signature was then defined as the product of CD8+ and PD-L1+ cell densities in the annotated tumor region and a cutoff of 1.54 × 10^5^ cells^2^/mm^4^ for signature positivity was applied. The CD8xPD-L1 signature, its two individual components, and PD-L1 expression (TC ≥25%) assessed manually were applied to the training, test and combined set of durvalumab-treated patients from Study 1108, as well as to the independent set of non-IO treated patients.

### Statistical analysis

PPV was calculated by dividing the number of true positive patients (signature-positive patients who showed either complete or partial clinical response according to RECIST v1.1) by the number of all signature-positive patients. This is equivalent to the ORR within the signature-positive subgroup. With the exception of PD-L1 TC ≥25%, the cutoffs for all signatures were defined by optimizing for PPV on the training set, while applying constraints on log-rank test-derived *p*-values for OS and PFS (≤ 0.05) and on the prevalence of signature-positive patients between 30 and 70% (Additional file 1: Figure S3). Once determined, only a single cutoff each for CD8+ cells, PD-L1+ cells, and the CD8xPD-L1 signature was used for the various performance parameters shown later. The CD8xPD-L1 signature was tested on the non-ICT NSCLC patient set by matching the prevalence of all signature-positive durvalumab-treated patients across both the training and test sets (36%). Accordingly, the non-ICT patients were ranked by their CD8xPD-L1 scores and the top 36% were considered to be CD8xPD-L1 signature-positive.

Multivariate Cox regression analysis [41–43] was performed on OS to provide a robust comparison between CD8xPD-L1 signature-negative and signature-positive patients in the context of clinically used strata: histology, smoking status, age, gender, liver metastasis, tumor stage and previous lines of therapy, each with their respective baseline values. Each of the tested measurements was added separately as a covariate to the set of fixed covariates, resulting in a set of eight covariates in total for Cox regression analysis. The analysis was performed on 163 patients with 98 events from Study 1108. A nested model approach was used to evaluate the product of CD8+ and PD-L1+ cell densities compared to the individual cell densities in the Cox model. The nested model of fitted objects was compared using an analysis of variance (ANOVA), giving an estimate of the difference between the respective models, indicated by the *p*-value. The *p*-values for the covariates in the Cox model and the ANOVA were considered significant if ≤0.05. Statistical calculations were performed using R version 3.4.2 with the Survival package 2.41–3 [44] and the Statistics package 3.4.2 [45].

## Results

### Durvalumab-treated patients, training set

At baseline, 31.0% of samples from patients enrolled in Study 1108 in the training set were CD8xPD-L1 signature-positive, 38.1% had high densities of CD8+ cells, 31.0% had high densities of PD-L1+ cells, and 58.3% had PD-L1 TC ≥25%. The CD8xPD-L1 signature provided the highest PPV (0.42), versus a high density of CD8+ cells (0.34), a high density of PD-L1+ cells (0.39), and PD-L1 TC ≥25% (0.29). For ORR, the CD8xPD-L1 signature was the only tested measure for which the PPVs for positive and negative patients had non-overlapping 95% confidence intervals (CIs): 0.42 (95% CI, 0.23–0.63) for signature-positive patients and 0.09 (95% CI, 0.03–0.19) for signature-negative patients. Additionally, CD8xPD-L1 signature-positive patients had significantly longer median OS (18.9 months [95% CI, 8.2–not reached; NR]) than signature-negative patients (8.9 months [95% CI, 4.1–12.9], *p* = 0.024) (Table 1 and Additional file 1: Figure S4A). In terms of the other tested measures, CD8+ cell density (Additional file 1: Figure S4B) demonstrated a statistically significant stratification of median OS (high: 18.9 months [95% CI, 12.9–NR]; low: 8.8 months [95% CI, 4.3–11.1], *p* = 0.012). Likewise, PD-L1 TC ≥25% (Additional file 1: Figure S4C) provided a statistically significant stratification of median OS (≥25%: 17.9 months [95% CI, 8.9–NR]; < 25%: 7.6 months [95% CI, 3.4–12.9], *p* = 0.0178). PD-L1+ cell density did not provide a significant stratification for OS (*p* = 0.071).

**Table 1:** Performance of the CD8xPD-L1 signature, its components, and manual PD-L1 scoring in durvalumab-treated patients

Measure	Group (*n*)	Prevalence	PPV (95% CI)	Median OS, months (95% CI)	OS *p-*value	Median PFS, months (95% CI)	PFS *p-*value
Training Set (*n* = 84)							
CD8xPD-L1	positive (26)	0.31	0.42 (0.23–0.63)	18.9 (8.2–NR)	0.024	5.3 (2.6–9.3)	0.00042
	negative (58)	0.69	0.09 (0.03–0.19)	8.9 (4.1–12.9)		1.4 (1.2–1.4)	
CD8+ cell density	high (32)	0.38	0.34 (0.19–0.53)	18.9 (12.9–NR)	0.012	4.4 (1.4–7.6)	0.00045
	low (52)	0.62	0.10 (0.03–0.21)	8.8 (4.3–11.1)		1.4 (1.2–2.3)	
PD-L1+ cell density	high (26)	0.31	0.39 (0.20–0.59)	18.9 (5.6–NR)	0.071	4.7 (1.6–7.6)	0.023
	low (58)	0.69	0.10 (0.04–0.21)	8.9 (4.1–13.1)		1.4 (1.3–1.7)	
PD-L1 TC	≥25% (49)	0.58	0.29 (0.17–0.43)	17.9 (8.9–NR)	0.018	2.8 (1.4–5.3)	0.0048
	< 25% (35)	0.42	0.06 (0.01–0.19)	7.6 (3.4–12.9)		1.4 (1.2–1.4)	
Test Set (*n* = 79)							
CD8xPD-L1	positive (33)	0.42	0.36 (0.20–0.55)	24.2 (14.5–NR)	0.00011	7.3 (3.1–9.8)	0.00095
	negative (46)	0.58	0.07 (0.01–0.18)	6.5 (4.2–9.8)		2.6 (1.4–3.9)	
CD8+ cell density	high (42)	0.53	0.24 (0.12–0.40)	20.3 (14.0–27.8)	0.0044	5.5 (3.1–9.2)	0.0054
	low (37)	0.47	0.14 (0.05–0.29)	6.5 (3.6–9.8)		2.5 (1.4–4.1)	
PD-L1+ cell density	high (29)	0.37	0.38 (0.21–0.58)	24.3 (6.5–NR)	0.045	7.3 (2.6–9.2)	0.087
	low (50)	0.63	0.08 (0.02–0.19)	9.3 (6.0–15.5)		2.8 (1.7–5.2)	
PD-L1 TC	≥25% (47)	0.59	0.28 (0.16–0.43)	15.5 (7.7–24.2)	0.19	4.8 (2.6–7.3)	0.25
	< 25% (32)	0.41	0.06 (0.01–0.21)	7.8 (5.7–15.5)		2.8 (1.4–6.5)	

*Abbreviations*: *CD8* Cluster of differentiation 8, *CI* Confidence interval, *NR* Not reached, *OS* Overall survival, *PD-L1* Programmed death ligand-1, *PFS* Progression-free survival, *PPV* Positive predictive value, *TC* Tumor cell

### Durvalumab-treated patients, test set

**Fig. 2:**
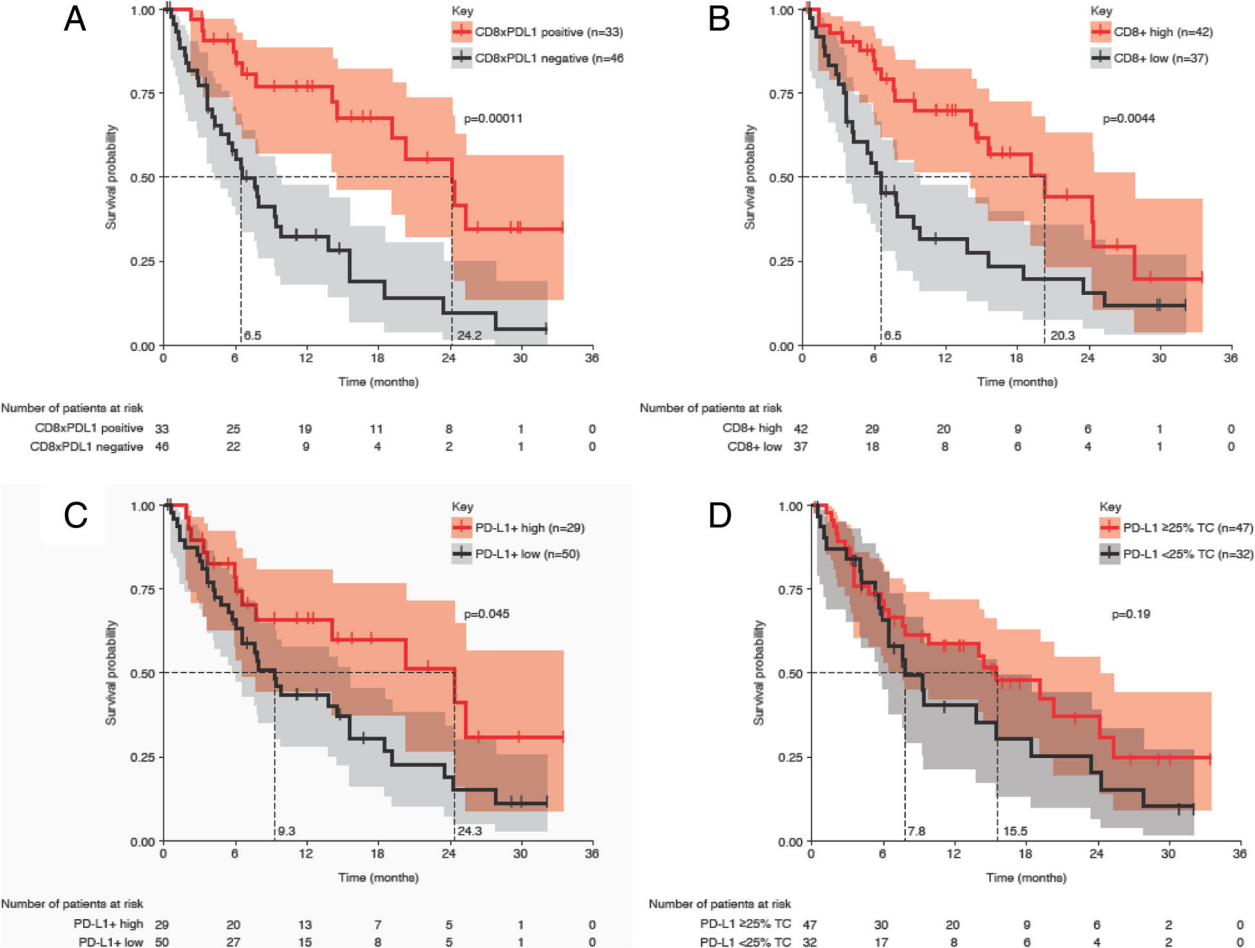
Predictive value of the CD8xPD-L1 signature compared to individual components. The comparative values are demonstrated by Kaplan-Meier analysis for overall survival of the durvalumab-treated patient test set for CD8xPD-L1 signature (**a**), CD8+ cell density (**b**), programmed cell death ligand-1 (PD-L1) + cell density (**c**), and manual pathologist scoring of PD-L1 tumor cell expression (**d**). Kaplan-Meier curves show survival probability, with shaded areas representing 95% confidence intervals. The cutoff values by which each measure was determined positive or negative were 1.54 × 10^5^ cells^2^/mm^4^ for CD8xPD-L1 signature positivity; 297 cells/mm^2^ for CD8+ tumor infiltrating lymphocyte density; and 644 cells/mm^2^ for PD-L1+ cell density. The cutoff value for PD-L1 manual scoring, ≥25% tumor cells, was determined previously [39]

After cutoff optimization on the training set, the respective signatures were applied to the test set of Study 1108 samples. The CD8xPD-L1 signature (Fig. 2a) again demonstrated the best stratification in terms of log-rank *p*-value compared to CD8+ cell density (Fig. 2b), PD-L1+ cell density (Fig. 2c) and PD-L1 TC ≥25% (Fig. 2d) (0.0001 versus 0.004, 0.045, and 0.19, respectively). Median OS was significantly longer in signature-positive patients (24.2 months [95% CI, 14.5–NR]) compared with signature-negative patients (6.5 months [95% CI, 4.2–9.8], *p* = 0.00011). Further, median OS was significantly longer for patients with high CD8+ cell density (20.3 months [95% CI, 14.0–27.8]) than for those with low density (6.5 months [95% CI, 3.6–9.8], *p* = 0.0044) and significantly longer in patients with high PD-L1+ cell density (24.3 months [95% CI, 6.5–NR]) than in those with low density (9.3 months [95% CI, 6.0–15.5], *p* = 0.045). Additionally, median OS was numerically longer in patients with PD-L1 TC ≥25% (15.5 months [95% CI, 7.7–24.2]) than in those with PD-L1 TC < 25% (7.8 months [95% CI, 5.7–15.5], *p* = 0.19) (Table 1). In terms of PFS, the only two tested measures that provided a statistically significant stratification were the CD8xPD-L1 signature (positive: 7.3 months [95% CI, 3.1–9.8]; negative: 2.6 months [95% CI, 1.4–3.9], *p* = 0.000945) and CD8+ cell density (high: 5.5 months [95% CI, 3.1–9.2]; low: 2.5 months [95% CI, 1.4–4.1], *p* = 0.00541) (Table 1).

Of note, PD-L1 scoring by automated image analysis provided better OS stratification than manual PD-L1 scoring in the test set. This may possibly reflect the incorporation of both TC and non-TC (primarily macrophage) PD-L1 expression in the automated image analysis scores versus the exclusion of immune cell PD-L1 expression in the manual scores. However, any advantage provided by automated image analysis compared to manual PD-L1 scoring in terms of overall predictive value was not clear.

The multiparametric Cox analysis of OS showed that the CD8xPD-L1 signature in the test set had better predictive value than its single components, manual PD-L1 status and also the presence of liver metastasis. Additionally, it was the only statistically significant measure in terms of the overall Cox model (Table 2).

**Table 2:** Multiparametric Cox analysis of predictive signatures (test set)

Significant fixed covariate	Significance [fixed covariate]	Added covariate	Significance [added covariate]	Significance [Cox Model]
None	N/A	None	N/A	–
LM	+	PD-L1 expression	–	–
None	N/A	CD8+ cell density	+	–
LM	+	PD-L1+ cell density	+	–
None	N/A	CD8xPD-L1 signature	+++	++

The parameters shown represent an analysis performed on the test set of durvalumab-treated patient tumor samples. Fixed covariates at baselines were histology, smoking status, age, gender, liver metastasis, tumor stage and line of therapy. - > 0.05; + ≤ 0.05; ++ ≤ 0.005; +++ ≤ 0.0005

*Abbreviations*: *CD8* Cluster of differentiation 8, *LM* Liver metastasis, *N/A* Not applicable, *PD-L1* Programmed cell death ligand-1

### Durvalumab-treated patients, combined set

**Fig. 3:**
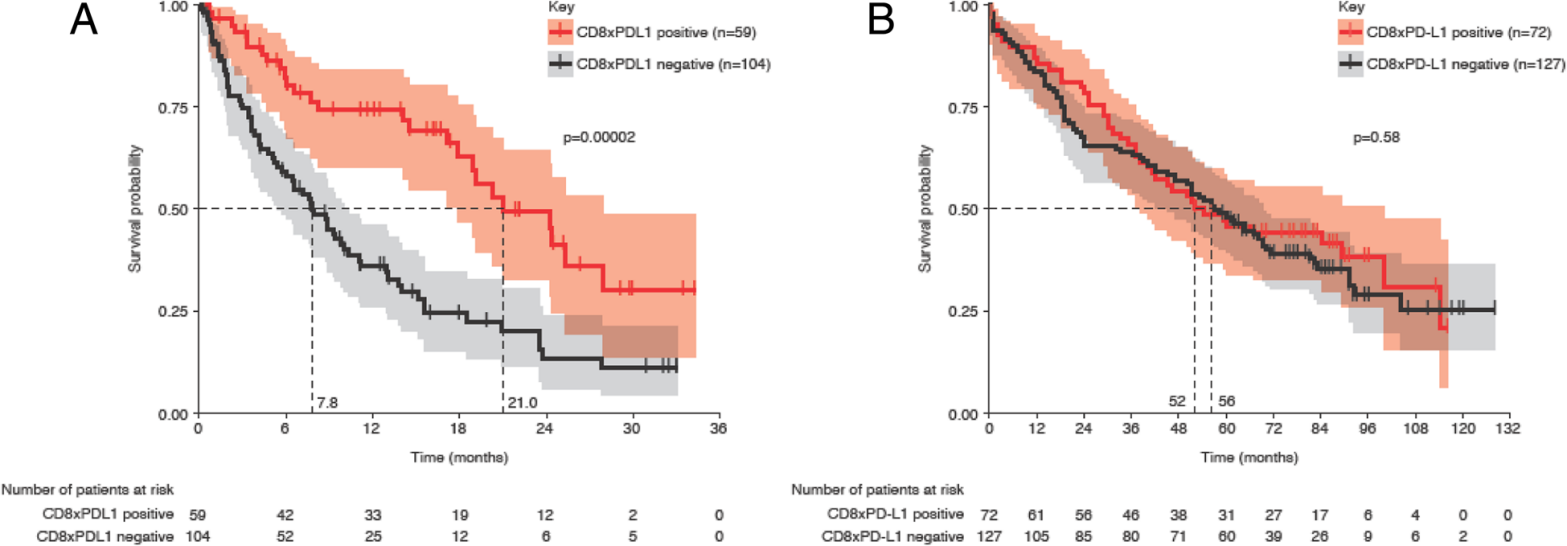
Predictive versus prognostic values of the CD8xPD-L1 signature. These are demonstrated by Kaplan-Meier analysis of overall survival for the CD8xPD-L1 signature in the combined (training and test) set of patients treated with durvalumab (**a**) compared to the set of non-immune checkpoint therapy (ICT) patients (**b**). Kaplan-Meier curves show survival probability, with shaded areas representing 95% confidence intervals. The prevalence for the non-ICT patients was matched to that for patients treated with durvalumab. The resulting cutoffs for CD8xPD-L1 signature positivity for the durvalumab and non-ICT sets respectively were 1.54 × 10^5^ and 2.85 × 10^4^ cells^2^/mm^4^

In the combined set of durvalumab-treated patients (Additional file 2: Table S2), the PPV for CD8xPD-L1 positivity was 0.39 and the PPV for high PD-L1+ cell density was 0.38; both were higher than those of PD-L1 TC ≥25% and high CD8+ cell density (both 0.28). For OS, CD8xPD-L1 demonstrated the strongest stratification of all tested measures, being significantly longer for signature-positive patients compared with signature-negative patients (21.0 months [95% CI, 17.9–27.9] versus 7.8 months [95% CI, 5.4–10.3], *p* = 0.00002) (Fig. 3a). Patients with high CD8+ cell density demonstrated statistically longer median OS compared with those with low density (20.3 months [95% CI, 15.5–24.3] versus 7.6 months [95% CI, 5.1–9.8], *p* = 0.00013). Likewise, median OS was significantly longer in patients with high PD-L1+ cell density than in those with low density (20.3 months [95% CI, 14.0–27.9] versus 9.3 months [95% CI, 6.5–13.1], *p* = 0.0064) and was significantly longer in patients with PD-L1 TC ≥25% than in those with PD-L1 < 25% (17.9 months [95% CI, 10.3–24.2] versus 7.8 months [95% CI, 6.0–11.1], *p* = 0.0082) (Additional file 1: Figure S5 and Additional file 2: Table S2). All four tested measures were associated with statistically significant stratifications for PFS (Additional file 2: Table S2).

The multiparametric Cox analysis showed that the CD8xPD-L1 signature was significantly and independently associated with OS for patients treated with durvalumab and had improved value in predicting OS compared with its single components, manual PD-L1 status and the presence of liver metastasis (Additional file 2: Table S3). Significant OS benefit was observed in CD8xPD-L1 signature-positive patients compared with signature-negative patients, regardless of the presence of liver metastases. The median OS for patients with liver metastases (Additional file 1: Figure S6) was significantly shorter than that of patients without liver metastases (6.0 months [95% CI, 2.2–11.1] versus 15.5 months [95% CI, 9.4–20.9], *p* ≤ 0.005). However, in the subgroup of patients with liver metastases, CD8xPD-L1 signature-positive patients had significantly longer median OS than CD8xPD-L1 signature-negative patients (14.5 months [95% CI, 3.2–NR] versus 5.4 months [95% CI, 1.8–9.8], *p* ≤ 0.05). In contrast, there was no statistically significant difference in OS between patients with PD-L1 TC ≥25% and those with PD-L1 TC < 25% in the subgroup with liver metastases (9.8 months [95% CI, 2.5–15.5] versus 5.4 months [95% CI, 1.1–10.0], *p* = 0.3). In the subgroup of patients without liver metastases, the CD8xPD-L1 signature demonstrated greater stratification for OS (positive: 24.3 months [95% CI, 17.9–NR]; negative: 8.9 months [95% CI, 6.5–14.0], *p* = 0.0002) than PD-L1 TC expression (TC ≥25%: 20.3 months [95% CI, 14.0–NR]; < 25%: 8.7 months [95% CI, 6.4–15.1], *p* = 0.008). A nested model approach was performed to further test if the CD8xPD-L1 signature would provide added predictive value to a model composed of its individual components. The model consisted of a reduced set of cofactors compared to the Cox models; liver metastasis, CD8+ cell density, and PD-L1+ cell density were compared to an identical model that contained CD8xPD-L1 as an additional cofactor. Other cofactors did not show significant contribution in the Cox models; they were therefore not considered for this comparison. CD8xPD-L1 significantly contributed to the model (*p* = 0.025).

### Non-ICT patients

**Fig. 4:**
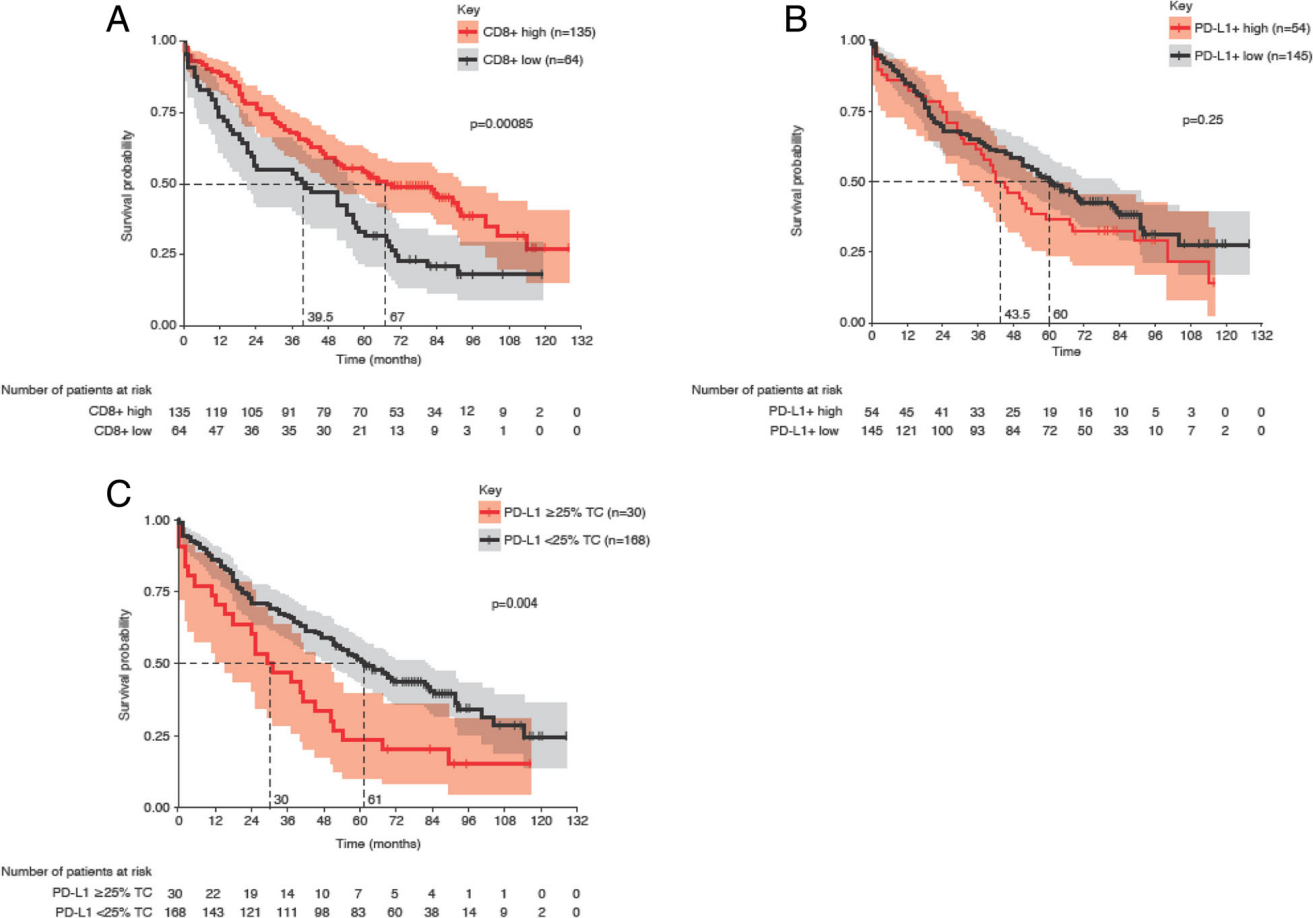
The prognostic values of CD8+ tumor infiltrating lymphocyte (TIL) densities and programmed cell death ligand-1 (PD-L1) measures. These are demonstrated by Kaplan-Meier analysis for overall survival by CD8+ (**a**) and PD-L1+ (**b**) cell densities and manual pathologist scoring of PD-L1 tumor cell expression (**c**) in patients who did not receive immune checkpoint therapy. Kaplan-Meier curves show survival probability, with shaded areas representing 95% confidence intervals. The cutoff values by which each measure was determined positive or negative were 297 cells/mm^2^ for CD8+ TIL density and 644 cells/mm^2^ for PD-L1+ cell density. The cutoff value for PD-L1 manual scoring, ≥25% tumor cells, was determined previously [39]

The CD8xPD-L1 signature was found not to be prognostic in the non-ICT setting. The median OS from the time of surgery for signature-positive patients was 52 months (95% CI, 37–89) versus 56 months (95% CI, 42–69) for signature-negative patients (Fig. 3b and Additional file 2: Table S2). However, a high density of CD8+ cells was associated with prolonged OS. The median OS from the time of surgery for patients with high CD8+ cell density was 67 months (95% CI, 50–92) versus 39.5 months (95% CI, 21–56) for patients with low density (*p* = 0.00085) (Fig. 4a and Additional file 2: Table S2). PD-L1+ cell density was not predictive of OS in the non-ICT group when the cutoff was transferred by prevalence matching (see Statistical analysis). The PD-L1 manual score TC ≥25% was significantly associated with poor OS (*p* = 0.004).

## Discussion

To date, the search for predictors of patient response to PD1/PD-L1 blockade has focused mainly on tumoral PD-L1 expression assessed manually via IHC, TMB, interferon-γ gene-related signatures, and CD8 analysis. We identified an automated image analysis signature comprised of PD-L1+ and CD8+ cell densities in tumor biopsies that predicts response to durvalumab monotherapy in patients with NSCLC. Multiple characteristics of this CD8xPD-L1 signature showed that it predicts response better than manual PD-L1 IHC scoring (TC ≥25%), which is the current benchmark for profiling patients most likely to respond to anti-PD1 and anti-PD-L1 immunotherapies. We also evaluated the individual components in comparison to manual PD-L1 scoring and further investigated the prognostic value of these measures in patients with NSCLC treated with non-ICT, which further supported the predictive value of the CD8xPD-L1 signature. This signature offers a number of advantages compared with current means of predicting response to anti-PD1 and anti-PD-L1 immunotherapies.

We tested the predictive benefit of CD8xPD-L1 signature in tumor samples of NSCLC patients enrolled in a Phase 1/2 study of durvalumab (Study 1108). Following optimization in a training set of samples, in the test set we found that the CD8xPD-L1 signature provided significant stratification for ORR, with non-overlapping 95% CIs between the PPVs for signature-positive and signature-negative patients. Additionally, it was the only statistically significant measure in the overall Cox model in the test set. In the combined set of samples from patients treated with durvalumab, the CD8xPD-L1 signature also demonstrated greater stratification for OS compared to PD-L1+ cell density, CD8+ cell density and manual PD-L1 TC ≥25%. These results were irrespective of liver metastasis status, although patients with liver metastases had significantly shorter median OS than those without. However, even in patients with liver metastasis, CD8xPD-L1 better identified patients with longer survival, as it provided improved stratification in terms of median OS compared with PD-L1 TC ≥25%.

After demonstrating the improvement of CD8xPD-L1 over PD-L1+ and CD8+ cell densities individually and manual PD-L1 TC ≥25% in predicting OS in durvalumab-treated patients, we tested the signature in a Cox model. A nested approach showed that the inclusion of CD8xPD-L1 as a covariate in addition to its single components and liver metastasis status resulted in a model statistically different from the same model lacking CD8xPD-L1, further reinforcing the predictive value of the signature.

Separately, we applied the CD8xPD-L1 signature to a set of surgically resected NSCLC tumor specimens from patients who received chemotherapy but not ICT to assess its prognostic effect. The signature did not stratify for OS, providing additional support for its utility as a predictive factor for durvalumab. However, we did find that CD8+ cell density alone provided statistically significant stratification of OS in patients not receiving ICT. These findings are consistent with other lines of evidence pointing to the prognostic value of tumoral CD8 status [25, 35, 46]. Our findings are also consistent with a study showing that a combination of TILs and PD-L1 expression was not prognostic for survival in patients with early stage resectable NSCLC [31], although another study found prognostic value in a combination of CD8+ TILs and PD-L1 expression in a similar population [46].

While the predictive value of the CD8xPD-L1 signature appears promising in this regard, a number of signature-positive patients did not respond to durvalumab. However, this limitation is not unlike the imperfect predictive value attributed to other assays of ICT, namely TMB and interferon-γ gene-related signatures [17–22], as well as PD-L1 manual scoring. Despite their limitations, the relative merits of TMB and interferon-γ gene-related signatures compared with PD-L1 IHC have become the subject of discussion recently. For example, the interferon-γ gene-related signature has shown predictive value independent of PD-L1 IHC status in patients receiving durvalumab, whereas TMB has shown predictive value irrespective of PD-L1 status in patients treated with nivolumab plus ipilimumab [20, 23]. However, comparisons of PD-L1 IHC with these markers are problematic due to a reliance on manually determined PD-L1 TC expression, as well as the use of different cutoff values for positivity that may not reflect the complexity of even this single biomarker. The effects of sampling error and the use of multiple assays for PD-L1 IHC, as well as for TMB and interferon-γ gene-related signatures, represent further complications that make the interpretation of these comparisons difficult. Nonetheless, these studies provide evidence that PD-L1 IHC, TMB, and interferon-γ demonstrate a degree of overlap in some patients. For instance, we showed that interferon-γ gene expression was associated with TMB in both NSCLC and urothelial carcinoma patients [23], and separately that the prevalence of patients with high levels of PD-L1+ cells as well as CD8+ cells, based on a different image analysis measure than that reported here, correlated with TMB across multiple tumor types [47]. Despite the challenges of directly comparing these biomarkers and the recognized limitations of PD-L1 IHC, there is support for the notion that PD-L1 expression may have predictive value, especially in combination with other tumoral measures. Here, we specifically show the value of PD-L1 expression combined with CD8+ TILs, thus combining measures of neoplastic cell characteristics and immune contexture. Combining additional biomarkers might provide further predictive value for cancer patients undergoing ICT.

Automated image analysis applied to IHC biomarkers provides potential advantages over manual scoring in the clinical setting. This is especially important in the case of manual IHC assessment of PD-L1 expression, where inter-observer variability in scoring has been reported in multiple studies [36, 48, 49]. Whereas our automated image analysis method measures PD-L1 in the entire annotated tumor region as a continuous variable, manual scoring by pathologists provides only a visual estimate of PD-L1 expression in the same tumor region. Such scoring results are often represented in terms of a scaled or categorical system [36, 48]. Thus, digital assessment has the ability to provide relatively greater accuracy and reproducibility across a range of tumor samples than manual assessment, especially at low levels of PD-L1 expression where inter-pathologist concordance has been deemed more problematic [36, 48]. Another potential problem related to PD-L1 IHC is the difficulty of combining PD-L1 expression in neoplastic and immune cells, primarily macrophages, because manual scoring of these distinct cell compartments is fundamentally different [6, 39, 48, 49]. The digital PD-L1 scoring used in this study combines the neoplastic and immune cell compartments into a single density score. We did observe some improved performance measures of the image analysis PD-L1 scores compared to manual PD-L1 scoring, though any advantage of automated image analysis, in terms of predictive value alone, was not clear. Importantly, neither the density of PD-L1+ cells as measured by automated image analysis nor the PD-L1 tumor cell score assessed by pathologists provided the predictive power of the CD8xPD-L1 signature, demonstrating the value of adding information on the presence of CD8+ T-cells to the patient stratification decision.

Furthermore, automated image analysis becomes of even greater value in the setting of combined IHC markers, where assessment of individual markers across the entire tumor region needs to be coordinated. As we demonstrated here and previously [37], this is the case whether the combined markers are quantified through the use of multiplex labeling of individual tissue sections or co-registration of single-stained serial sections. It should also be recognized that analysis of tumors histologically to assess the immune response to cancer is trending toward increased reliance on multiplex immunofluorescence that labels many relevant immune markers. As illustrated recently [22], the complex information made evident in this approach overwhelms the ability to quantify marker-positive cells in entire tumor samples manually. Digital analysis is therefore necessary to obtain the greatest value from this approach. Quantifying the spatial relationships between various types of immune cells or otherwise quantifying the complexity of the tumor microenvironment based on multiplex immunolabeling will further require the use of image analysis. Computational histological assessment also has the potential to extract other types of information from tumor biopsies, as demonstrated in a recent study that used automated image analysis of routinely stained tissues to predict tumor mutational changes in NSCLC [50]. This kind of information could be combined with digital markers such as the CD8xPD-L1 signature reported here or with a variety of other markers to continue to improve precision medicine approaches for ICT.

## Conclusions

We successfully developed an automated digital signature based on the product of the densities of CD8+ cells and PD-L1+ cells measured by automated image analysis applied to consecutive IHC-stained lung cancer tissue sections. This signature resulted in significantly greater stratification of survival for patients with NSCLC treated with durvalumab than CD8+ TIL density, PD-L1+ cell density or manually derived PD-L1 expression alone. This demonstrates the predictive value of accounting for both tumor factors (PD-L1) and immune contexture in profiling cancer patients for response to ICT. Liver metastasis was the only other covariate considered to significantly contribute to the model. This study also showed that computational analysis of routine tumor specimens can be practically applied to large sets of clinical trial and non-clinical sample biopsies in a manner with multiple advantages over manually derived means. This approach, therefore, may offer a foundation for the development of companion diagnostic tests of even greater complexity to select patients most likely to respond to ICT targeting the PD1/PD-L1 axis with greater precision than current methods. Nonetheless, the predictive value of the CD8xPD-L1 signature requires verification in additional studies.

## Additional files

Additional file 1: **Figure S1** Digital image analysis segments CD8+ and PD-L1+ cells in dual-labelled sections of NSCLC. **Figure S2.** Channel unmixing used to segment CD8+ and PD-L1+ cells in CD8/PD-L1 dual chromogenic immunohistochemistry assay. **Figure S3.** Optimization of cutoff values for CD8xPD-L1 signature, CD8+ cell density, and PD-L1+ cell density was performed on the training set of samples of durvalumab-treated patients. **Figure S4.** The predictive value of the CD8xPD-L1 signature compared to its individual components in training sample set. **Figure S5.** Predictive values of the individual components of the CD8xPD-L1 signature in the combined sample set. **Figure S6.** Analysis of OS for the CD8xPD-L1 signature and liver metastasis. (DOCX 990 kb)

Additional file 2: **Table S1.** Patient demographics and baseline characteristics for analysed samples. **Table S2.** Performance of the CD8xPD-L1 signature, its individual components, and PD-L1 TC expression in the combined set of durvalumab-treated and non-ICT-treated patients. **Table S3.** Multiparametric Cox analysis of signatures in the entire data set including additional PD-L1 readouts. (DOCX 28 kb)

## Abbreviations

ANOVA: Analysis of variance
CD8: Cluster of differentiation 8
CI: Confidence interval
ICT: Immune checkpoint therapy
IHC: Immunohistochemistry
NR: Not reached
NSCLC: Non-small cell lung cancer
ORR: Objective response rate
OS: Overall survival
PD1: Programmed cell death-1
PD-L1: Programmed cell death ligand-1
PFS: Progression-free survival
PPV: Positive predictive value
RECIST: Response Evaluation Criteria in Solid Tumors
TC: Tumor cell
TIL: Tumor infiltrating lymphocyte
TMB: Tumor mutational burden
