# Supplementary material for: Automated image analysis of NSCLC biopsies to predict response to anti-PD-L1 therapy
Source: J Immunother Cancer. 2019 May 6;7:121. doi: 10.1186/s40425-019-0589-x (PMC6501300; doi:10.1186/s40425-019-0589-x)
Supplement: Supplementary file 1 — Figure S1 Digital image analysis segments CD8+ and PD-L1+ cells in dual-labelled sections of NSCLC. Figure S2. Channel unmixing used to segment CD8+ and PD-L1+ cells in CD8/PD-L1 dual chromogenic immunohistochemistry assay. Figure S3. Optimization of cutoff values for CD8xPD-L1 signature, CD8+ cell density, and PD-L1+ cell density was performed on the training set of samples of durvalumab-treated patients. Figure S4. The predictive value of the CD8xPD-L1 signature compared to its individual components in training sample set. Figure S5. Predictive values of the individual components of the CD8xPD-L1 signature in the combined sample set. Figure S6. Analysis of OS for the CD8xPD-L1 signature and liver metastasis. (DOCX 990 kb) [file 40425_2019_589_MOESM1_ESM.docx]

Automated image analysis of NSCLC biopsies to predict response to anti-PD-L1 therapy

Althammer S, Tan TH, Spitzmüller A, Rognoni L, Wiestler T, Herz T, Widmaier M, Rebelatto MC, Kaplon H, Damotte D, Alifano M, Hammond SA, Dieu-Nosjean M-C, Ranade K, Schmidt G, Higgs BW, Steele KE

**Additional file 1: Supplementary Figures**

**
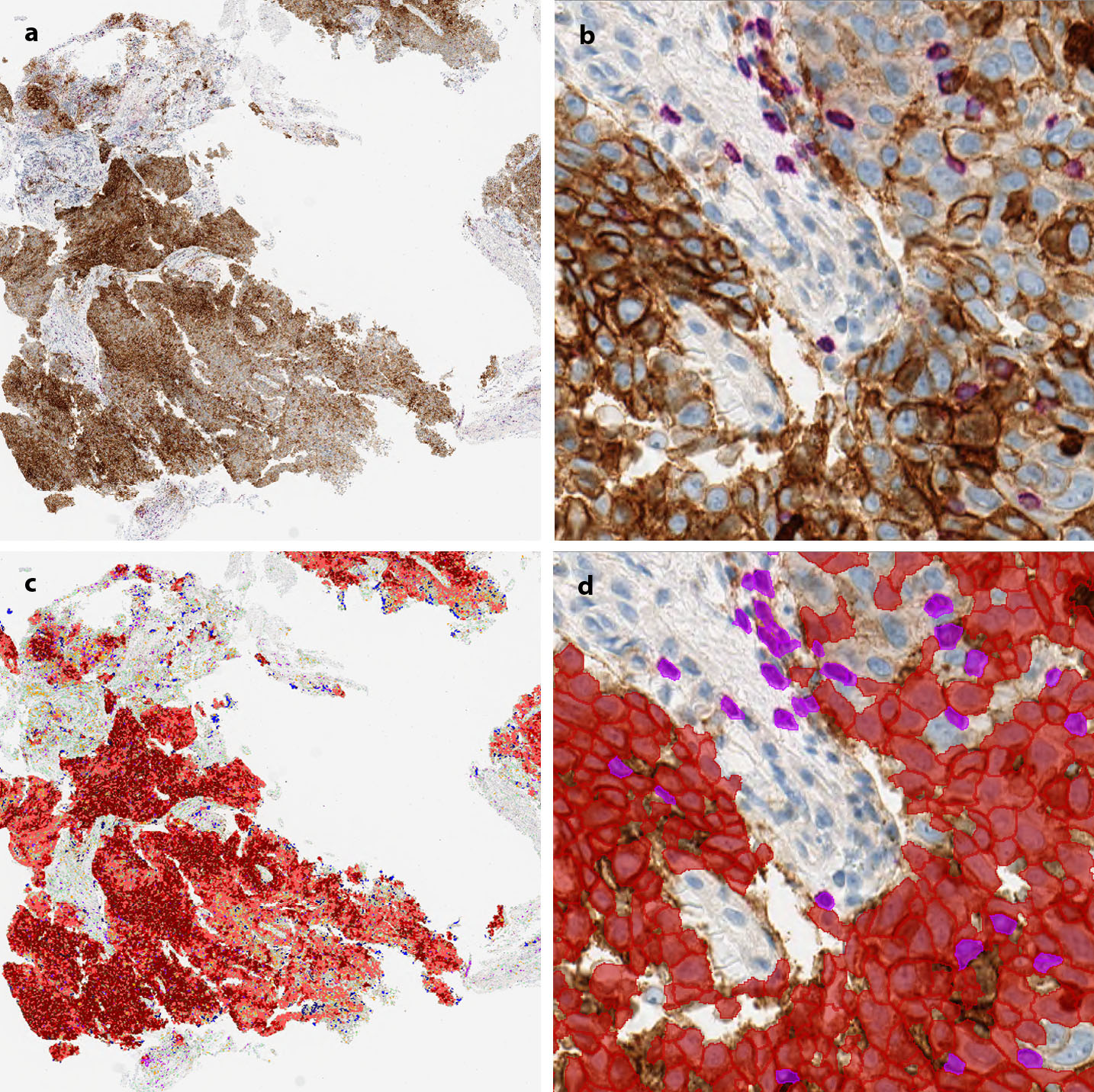
**

**Fig. S1.** Digital image analysis segmentation of CD8+ and programmed cell death ligand-1 (PD-L1)+ cells in a dual-labeled section of non-small cell lung cancer (NSCLC). A single tumor section of a screened NSCLC patient who was unenrolled in Study 1108 labeled with a dual immunohistochemistry assay using purple chromogen for CD8 and brown chromogen for PD-L1 (**a**, **b**). Image analysis segmentation (**c**, **d**) of CD8+ cells is shown as purple and PD-L1+ cells as red. At high magnification (**d**), segmentation of CD8+ tumor infiltrating lymphocytes in both PD-L1+ tumor islands and PD-L1-negative stroma is evident.

**
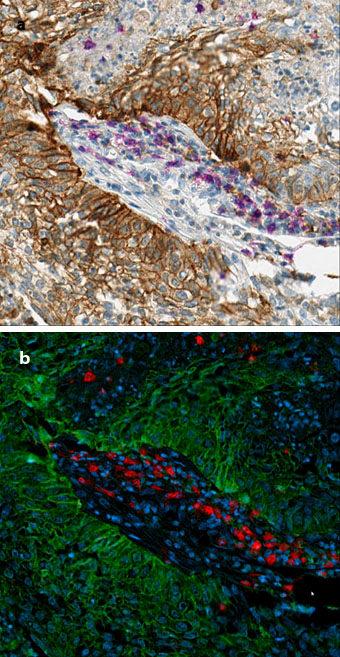
**

**Fig. S2**. Channel unmixing used to segment CD8 and programmed cell death ligand-1
(PD-L1) in the CD8/PD-L1 dual chromogenic immunohistochemistry (IHC) assay. A single tumor section from a patient with non-small cell lung cancer in the non-immune checkpoint therapy cohort was labelled with the dual IHC assay. The image analysis applied to the original image (a) uses channel unmixing to produce a false color representation (b), illustrating the program’s ability to digitally separate purple (CD8, pseudocolored red) from brown (PD-L1, pseudocolored green) chromogens. Light blue pseudocolor in b represents the hematoxylin counterstain.

**Fig. S3.** Optimization of cutoff values for CD8xPD-L1 signature (A), CD8+ cell density (B), and programmed cell death ligand-1 (PD-L1)+ cell density (C) was performed on the training set of samples of durvalumab-treated patients. Signature values were required to have a prevalence of between 30% and 70% in order to exclude low or non-representative numbers; the areas below or above these values are denoted in gray. The resulting cutoff numbers were: 1.54x10^5^ cells^2^/mm^4^ (CD8xPD-L1), 297 cells/mm^2^ (CD8+), and 644 cells/mm^2^ (PD-L1+).

**Fig. S4** The predictive value of the CD8xPD-L1 signature compared to its individual components in training sample set. The value of the CD8xPD-L1 signature is demonstrated by Kaplan-Meier analysis for overall survival of the durvalumab-treated patient training set as CD8xPD-L1 signature (A), CD8+ cell density (B), and programmed cell death ligand-1 (PD-L1)+ cell density (C). Kaplan-Meier curves show survival probability, with the shaded areas representing 95% confidence intervals. The cutoff values for positivity for each measure were 1.54x10^5^ cells^2^/mm^4^ for CD8xPD-L1 signature; 297 cells/mm^2^ for CD8+ tumor infiltrating lymphocyte density; and 644 cells/mm^2^ for PD-L1+ cell density.

**Fig. S5.** Predictive values of CD8xPD-L1 signature individual components in the combined sample set of durvalumab-treated patients. The values of CD8xPD-L1 signature individual components are demonstrated by Kaplan-Meier analysis for overall survival by CD8+ cell density (a), programmed cell death ligand-1 (PD-L1)+ cell density (b), and manual pathologist scoring of PD-L1 tumor cell expression (c). Kaplan-Meier curves show survival probability, with shaded areas representing 95% confidence intervals. The cutoff values for positivity were determined to be 297 cells/mm^2^ for CD8+ tumor infiltrating lymphoctye density and 644 cells/mm^2^ for PD-L1+ cell density. The cutoff value for PD-L1 manual scoring, ≥25% tumor cells, was determined previously [39]. The Kaplan-Meier curve for the CD8xPD-L1 signature in the combined set is shown in Fig. 3A.

**Fig. S6.** Analysis of overall survival by CD8xPD-L1 signature and presence of liver metastasis, assessed in the combined sample set of durvalumab-treated patients. The vertical green lines represent upper and lower bounds of 95% confidence intervals; for programmed cell death ligand-1 tumor cell ≥25% patients without liver metastases and signature-positive patients with or without liver metastases, there is no upper bound. Significance level of *p*-values: - > 0.05; + ≤ 0.05; ++ ≤ 0.005; +++ ≤ 0.0005; ++++ ≤ 0.00005.
