# Supplementary material for: Automated image analysis of NSCLC biopsies to predict response to anti-PD-L1 therapy
Source: J Immunother Cancer. 2019 May 6;7:121. doi: 10.1186/s40425-019-0589-x (PMC6501300; doi:10.1186/s40425-019-0589-x)
Supplement: Supplementary file 2 — Table S1. Patient demographics and baseline characteristics for analysed samples. Table S2. Performance of the CD8xPD-L1 signature, its individual components, and PD-L1 TC expression in the combined set of durvalumab-treated and non-ICT-treated patients. Table S3. Multiparametric Cox analysis of signatures in the entire data set including additional PD-L1 readouts. (DOCX 28 kb) [file 40425_2019_589_MOESM2_ESM.docx]

**Additional file 2**

**Table S1.** Patient demographics and baseline characteristics for analyzed samples.

|  | Durvalumab-treated cohort | | | | Non-ICT cohort  (n=199) |
| --- | --- | --- | --- | --- | --- |
|  | **Training set**  **(n=84)** | **Test set  (n=79)** | ***p*-value^a^** | **All  (n=163)** |  |
| **Sex (%)** |  |  | 0.55 |  |  |
| **F** | 46.4 | 41.8 |  | 44.2 | 20.1 |
| **M** | 53.6 | 58.2 |  | 55.8 | 79.9 |
| **Age (%)** |  |  | 0.06 |  |  |
| **≤35** | 1.2 | 1.3 |  | 1.2 | 0 |
| **36-45** | 2.4 | 3.8 |  | 3.1 | 6.5 |
| **46-55** | 8.3 | 25.3 |  | 16.6 | 18.1 |
| **56-65** | 34.5 | 26.6 |  | 30.7 | 23.6 |
| **66+** | 53.6 | 43.0 |  | 48.5 | 51.8 |
| **Smoking status (%)** |  |  | 0.90 |  |  |
| **0-14 pack-years** | 27.4 | 30.4 |  | 28.8 | 12.6 |
| **15+ pack-years** | 64.3 | 60.8 |  | 62.6 | 82.4 |
| **Unknown** | 8.3 | 8.9 |  | 8.6 | 5.0 |
| **Histology (%)** |  |  | 0.83 |  |  |
| **Squamous** | 53.6 | 51.9 |  | 52.8 | 38.7 |
| **Non-squamous** | 46.4 | 48.1 |  | 47.2 | 57.8 |
| **Other** | 0.0 | 0.0 |  | 0.0 | 3.5^b^ |
| **T stage (%)** |  |  | 0.88 |  |  |
| **T0** | 0.0 | 1.3 |  | 0.6 | 0 |
| **T1** | 11.9 | 12.7 |  | 12.3 | 22.6 |
| **T2** | 26.2 | 21.5 |  | 23.9 | 43.7 |
| **T3** | 13.1 | 16.5 |  | 14.7 | 23.6 |
| **T4** | 19.0 | 22.8 |  | 20.9 | 10.1 |
| **TX** | 9.5 | 8.9 |  | 9.2 | 0 |
| **Unknown** | 20.2 | 16.5 |  | 17.8 | 0 |
| **N stage (%)** |  |  | 0.43 |  |  |
| **N0** | 20.2 | 22.8 |  | 21.5 | 62.3 |
| **N1** | 14.3 | 5.1 |  | 9.8 | 19.1 |
| **N2** | 21.4 | 26.6 |  | 23.9 | 18.1 |
| **N3** | 16.7 | 20.3 |  | 18.4 | 0 |
| **NX** | 7.1 | 8.9 |  | 8.0 | 0.5 |
| **Unknown** | 20.2 | 16.5 |  | 17.8 | 0 |
| **M stage (%)** |  |  | 0.51 |  |  |
| **M0** | 28.6 | 31.6 |  | 30.1 | 100 |
| **M1** | 48.8 | 54.4 |  | 51.5 | 0 |
| **MX** | 3.6 | 1.3 |  | 2.5 | 0 |
| **Unknown** | 19.0 | 12.7 |  | 15.3 | 0 |
| **Cancer staging (%)** |  |  | 0.66 |  |  |
| **I** | 3.6 | 2.5 |  | 3.1 | 44.2 |
| **II** | 10.7 | 7.6 |  | 9.2 | 23.6 |
| **III** | 22.6 | 26.6 |  | 24.5 | 32.2 |
| **IV** | 61.9 | 60.8 |  | 61.3 | 0 |
| **Unknown** | 1.2 | 2.5 |  | 1.8 | 0 |
| **Line of therapy (%)** |  |  | 0.93 |  |  |
| **1** | 23.8 | 25.3 |  | 24.5 | 100 |
| **2** | 23.8 | 25.3 |  | 24.5 | 0 |
| **≥3** | 52.4 | 49.4 |  | 50.9 | 0 |
| **PD-L1 status (%)** |  |  | 0.99 |  |  |
| **<25%** | 41.7 | 40.5 |  | 41.1 |  |
| **≥25%** | 58.3 | 59.5 |  | 58.9 |  |
| **Response to durvalumab (%)^c^** |  |  | 0.09 |  |  |
| **CR** | 1.2 | 0.0 |  | 0.6 |  |
| **PR** | 17.9 | 19.0 |  | 18.4 |  |
| **SD** | 25.0 | 43.0 |  | 33.7 |  |
| **PD** | 41.7 | 24.1 |  | 33.1 |  |
| **NE** | 14.3 | 13.9 |  | 14.1 |  |

^a^*p*-value of a Chi-squared test comparing homogeneity between training and test sets. There is no significant difference observed between the training and test set for any of the clinical categories (all *p*-values > 0.05).

^b^Other NSCLC histology consist of carcinoma, not otherwise specified (n=2), and giant cell carcinoma (n=5).

^c^Response rates shown are for patients with suitable tumor specimens included in this analysis.

**Table S2.** Performance of the CD8xPD-L1 signature, its components, and manual PD-L1 TC scoring in the combined set of
durvalumab-treated and non-ICT patients.

| **Signature (n)** | **Set** | **Prevalence** | **Median OS, months**  **(95% CI)** | ***p*-value** | **PPV for response (95% CI)** | **Median PFS, months (95% CI)** | ***p*-value** |
| --- | --- | --- | --- | --- | --- | --- | --- |
| **CD8xPD-L1 positive (59)** | **Durvalumab** | 0.362 | **21.0  (17.9–27.9)** | **0.00002** | **0.39  (0.265–0.526)** | 7.3  (4.1–9.2) | 0.00000027 |
| **CD8xPD-L1 negative (104)** | **Durvalumab** | 0.638 | **7.8  (5.4–10.3)** |  | **0.0769  (0.0338–0.146)** | 1.4  (1.4–2.3) |  |
| **CD8+ high cell density (74)** | **Durvalumab** | 0.454 | **20.3  (15.5–24.3)** | **0.00013** | **0.284**  **(0.185–0.401)** | *5.3*  (3.1–7.4) | 0.00000192 |
| **CD8+ low cell density (89)** | **Durvalumab** | 0.546 | **7.6**  **(5.1–9.8)** |  | **0.112**  **(0.0552–0.197)** | 1.7  (1.4–2.6) |  |
| **PD-L1+ high cell density (55)** | **Durvalumab** | 0.337 | **20.3  (14.0–27.9)** | **0.0064** | **0.382  (0.254–0.523)** | 5.3  (2.8–7.4) | 0.00231 |
| **PD-L1+ low cell density (108)** | **Durvalumab** | 0.663 | **9.3  (6.5–13.1)** |  | **0.0926  (0.0453–0.164)** | 1.7  (1.4–2.8) |  |
| **PD-L1 TC ≥25% (96)** | **Durvalumab** | 0.589 | **17.9  (10.3–24.2)** | **0.0082** | **0.281  (0.194–0.382)** | 3.6  (2.6–5.3) | 0.00411 |
| **PD-L1 TC <25% (67)** | **Durvalumab** | 0.411 | **7.8  (6.0–11.1)** |  | **0.0597  (0.0165–0.146)** | 1.4  (1.3–2.6) |  |
| **CD8xPD-L1 positive (72)** | **Non-IO** | 0.362 | **52**  **(37–89)** | **0.58** | **NA**  **(0–1)** | NA | NA |
| **CD8xPD-L1 negative (127)** | **Non-IO** | 0.638 | **56**  **(42–69)** |  | **NA**  **(0–1)** | NA |  |
| **CD8+ high cell density (135)** | **Non-IO** | 0.678 | **67**  **(50–92)** | **0.00085** | **NA**  **(0–1)** | NA | NA |
| **CD8+ low cell density (64)** | **Non-IO** | 0.322 | **39.5**  **(21– 56)** |  | **NA**  **(0–1)** | NA |  |
| **PD-L1+ high cell density (54)** | **Non-IO** | 0.271 | **43.5**  **(31–59)** | **0.25** | **NA**  **(0–1)** | NA | NA |
| **PD-L1+ low cell density (145)** | **Non-IO** | 0.729 | **60**  **(47–81)** |  | **NA**  **(0–1)** | NA |  |
| **PD-L1 TC ≥25% (30)** | **Non-IO** | 0.152 | **30**  **(15–50)** | **0.004** | **NA**  **(0–1)** | NA | NA |
| **PD-L1 TC <25% (168)** | **Non-IO** | 0.848 | **61**  **(51–82)** |  | **NA**  **(0–1)** | NA |  |

**Table S3.** Multiparametric Cox analysis of signatures in the entire data set including additional PD-L1 readouts.

| **Significant fixed covariate** | **Significance (fixed covariate)** | **Added covariate** | **Significance (added covariate)** | **Significance (Cox model)** |
| --- | --- | --- | --- | --- |
| **Liver metastasis/line of therapy** | ++/+ | **None** | N/A | + |
| **LM** | +++ | **CD8xPD-L1 signature** | ++++ | ++++ |
| **LM** | + | **CD8+ cell density** | ++ | ++ |
| **LM** | ++ | **PD-L1 TC expression** | + | + |
| **LM** | +++ | **PD-L1+ cell density** | + | ++ |
| **LM** | +++ | **% of PD-L1+ pixels^a^** | + | ++ |
| **LM** | +++ | **% of PD-L1+ cells^b^** | + | ++ |

Fixed covariates at baseline were histology, smoking status, age, gender, liver metastasis, tumor stage and line of therapy. + ≤ 0.05; ++ ≤ 0.005; +++ ≤ 0.0005; ++++ ≤ 0.00005.
^a^Percentage of PD-L1+ pixels: Ratio of PD-L1+ pixels to total pixels in tissue
^b^Percentage of PD-L1+ cells: Ratio of PD-L1+ cells to all cells
